# Supplementary material for: Chromothripsis during telomere crisis is independent of NHEJ, and consistent with a replicative origin
Source: Genome Res. 2019 May;29(5):737–49. doi: 10.1101/gr.240705.118 (PMC6499312; doi:10.1101/gr.240705.118)
Supplement: Supplemental Material [file supp_gr.240705.118_Supplemental_file_1.zip › contigs/annotated_contigs/DB48/contig.7.DB48_length_1459_mean_cov_10.1446196025.docx]

**DB48_length_1459_mean_cov_10.1446196025**

```
TTATTTTTCTTTTAATTAAGAGAGCTGTGAAATTATTACCTATAATTCTAAATCTCATTTAATTCCTCCCAATAGGATTCAAGATGGAT
 >chr16:53405048-53405306 - E=5e-143 p=2e-02
TGTCATCAAAGTTCACTTCTTTAACAAAAGTGGTTTATGACTTTAATAAAATATTAGAGAATGGAAGGATCTGTGGAAGCCCTTTACAA

AAACTTGTGATAGAAAGTTTTGATGATGAGCAGACTTTGCAACAACTGGAATTGCAAAATGAACCAATTTTACAATGC|TT|TTATTTT
                                                                               >chr16:5
AGTCAGTTAAAAGTTCCAGAAATGTGAACAACAAAGATTTTTTTGATCCAGTTGAAAGTGATGAAGACATAGCAAGTGATCATGATGAT
3403824-53404218 - E=9e-222
GAGCTGGGTTCAAACAAGATGATGAAATTGCTGAAGAAGAAGCAGAAGAAGGAAGCATTTCTGAAATGTGAGTATTTTGAAACATCCTT

TACTTTGTGAGCTGGAATTGCCCAATCAGTGTTTGTATTTGTGGTTTTCACATATGTTTGTTTTAAGAAGTTAGATACTCTCCTATCAG

ATCTTCTCAAGATAGCCAGAGGAAAGTCACTGGATTTAAACGGACATTAGAGATCATTTAATGAAGAAAAATATTACTGACAGTAGCAA

TCAAACCTTTCTTACCAGGAACTTTG|ATT|ATAAGAACAACTGGAACATAAACTGAATGGAAGTACAGATTCATTTATAACTGATAAA
                           >chr16:53404371-53404620 + E=1e-137
ACAAAGCATAATATATCTTAGCTCTAAACCTTCTAACTACAATTACATCTCTCCTAGAAAAACAGAACAAAAGCTAATTTGAGGAGGAG

GAAAGTGCTCTCTCCTTTCTCAAACTTAACCTTAAGTTTTTTACTTCCAAACAGTCCCCTTTTATCTTCATCAGAATCAATATCTTCAA

AAAAAAATC|A|TAGATTGATGAATAATTTATTTTCTATATATTGGACCCTTTTTGTTTCTTACATAGCATTTCACTTAAATGATAGCT
          >chr16:53403659-53403771 - E=3e-55
TCTCTTCCTTAATACCTGAATGATTTTGGAAG|TT|ACAGGAGGAAGAGGAAGTGTCAGATATGGGTGGTGACAATCCTGAAGTGGGCA
                                 >chr16:53404672-53404928 - E=5e-137
AGAAAGCTAGAAACTCAAGCAAATTTGAGCTGAGGAAAAGCCCAGTTTCAGTGATGAGGATTCTGACCTTGACTTTGATATCAGCAAAT

TGGAACAGCAGAGCAAGGTGCAAAACACAGGACATGGAAAACCAAGAGAAAAGTCCATAATAGACGAGAAATTCTTCCAACTCTCTGAA

ATGGAGGCTTATTTAGAAAAC|A|AATCAGTATCTTCCACCTCATCATCATTATCATCTTTTCGTTCCTCTTCTTTTTCTCTGTTTTCT
                      >chr16:53404614-53404690 + E=1e-33 p=4e-02
AAATAAG|CCT|GTTATTTTTGTGAGATAAGAGGTTTGTATTATAGATTGATGAATAATTTATTTTCTATATATTGGACCCTTTTTGTT
        >chr16:53403679-53403804 - E=5e-63
TCTTACATAGCATTTCACTTAAATGATAGCTTCTCTTCCTTAATA|TC
```
